# Early Thymectomy Is Associated With Long-Term Impairment of the Immune System: A Systematic Review

**DOI:** 10.3389/fimmu.2021.774780

**Published:** 2021-11-25

**Authors:** Nara Vasconcelos Cavalcanti, Patrícia Palmeira, Marcelo Biscegli Jatene, Mayra de Barros Dorna, Magda Carneiro-Sampaio

**Affiliations:** ^1^ Children’s Hospital, Hospital das Clínicas da Faculdade de Medicina da Universidade de São Paulo (HC-FMUSP), São Paulo, Brazil; ^2^ Laboratory of Clinical Investigation LIM-36, Hospital das Clínicas da Faculdade de Medicina da Universidade de São Paulo (HC-FMUSP), São Paulo, Brazil; ^3^ Pediatric Cardiovascular Surgery Department, Heart Institute, Hospital das Clínicas da Faculdade de Medicina da Universidade de São Paulo (HC-FMUSP), São Paulo, Brazil

**Keywords:** thymus, thymectomy, congenital heart defect, lymphocytopenia, T lymphocyte, TRECs, T cell receptor repertoire, immunosenescence

## Abstract

**Background and Aims:**

Congenital heart diseases (CHDs) are diagnosed in approximately 9 in 1,000 newborns, and early cardiac corrective surgery often requires partial or complete thymectomy. As the long-term effect of early thymectomy on the subsequent development of the immune system in humans has not been completely elucidated, the present study aimed to evaluate the effects of thymus removal on the functional capacity of the immune system after different periods.

**Methods:**

A systematic review of the literature was performed using MEDLINE, EMBASE, LILACS and Scopus. The inclusion criteria were original studies that analyzed any component of the immune system in patients with CHD who had undergone thymectomy during cardiac surgery in the first years of life. The results were evaluated for the quality of evidence.

**Results:**

Twenty-three studies were selected and showed that patients who underwent a thymectomy in the first years of life tended to exhibit important alterations in the T cell compartment, such as fewer total T cells, CD4+, CD8+, naïve and CD31+ T cells, lower TRECs, decreased diversity of the TCR repertoire and higher peripheral proliferation (increased Ki-67 expression) than controls. However, the numbers of memory T cells and Treg cells differed across the selected studies.

**Conclusions:**

Early thymectomy, either partial or complete, may be associated with a reduction in many T cell subpopulations and TCR diversity, and these alterations may persist during long-term follow-up. Alternative solutions should be studied, either in the operative technique with partial preservation of the thymus or through the autograft of fragments of the gland.

**Systematic Review Registration:**

Prospero [157188].

## Introduction

Congenital heart diseases (CHDs) are diagnosed in approximately 9 of every 1,000 newborns, and one-third of these newborns have critical conditions requiring surgical treatment in the first years of life (1, 2). As the thymus obstructs the surgeon’s access during the procedure, it is frequently removed, either completely or partially (3). The removal of the thymus in early life may affect the subsequent development of the immune system in humans and therefore should be investigated in depth.

Historically, most patients with CHD died in early childhood; however, the past four decades have witnessed extraordinary advances in early diagnosis and cardiothoracic surgery, leading to an increased survival of newborns with CHD. In high-income countries, over 90% of children with CHD now survive into their adult years (4). Therefore, as adults with CHD age, the risk of complications increases and requires a better understanding of their ongoing needs (5).

From this perspective, the effect of early thymectomy has been the subject of research, but a significant clinical effect has not been described during the three decades of follow-up of thymectomized patients to date (6, 7), despite the major repercussions reported in murine models since the 1960s (8). However, laboratory evaluation has documented some concerning signs regarding immune system functioning since the first description by Brearley et al. in 1987 of significantly lower levels of T cells in thymectomized children than in controls (3). Since then, Immunology has evolved substantially, and new laboratory techniques have been developed. For instance, new cell subpopulations, such as regulatory T cells (Tregs), and new elements, such as T cell receptor excision circles (TRECs), were identified.

More recent studies have analyzed the effects of thymectomy after cardiac surgery by considering each of the following aspects: elements of the immune system, age when thymectomy was performed and the time span after thymectomy. Additionally, complete *versus* partial removal of the thymus might exert a different degree of effect (6, 7, 9, 10).

Contradictory findings have been reported. For example, total T cell, CD4^+^ and CD8^+^ T cell populations were found to be reduced or similar in thymectomized patients compared to controls (9, 10). Some studies have also shown that the levels of T cells and their subsets vary according to the time since thymectomy was performed (7, 10).

Relevant research has been performed; however, researchers have not yet clearly determined how and what factors influence the effect of early thymectomy on the immune system. Therefore, the aim of the present study was to compile the literature and evaluate the effects of thymectomy on the development and functional capacity of the immune system after different periods.

## Methods

### Study Design and Registry

A systematic review of the literature was performed according to the methodology established by the Preferred Reporting Items for Systematic Reviews and Meta-Analyses (PRISMA) (11) and the Meta-analysis Of Observational Studies in Epidemiology (MOOSE) group (12). The study was registered in the International Prospective Register of Systematic Reviews (PROSPERO) under code 157188.

### PICOS Strategy (Population, Interventions, Comparators, Outcomes, and Study Design)

The PICOS strategy was used to build the research question as follows:

Population: infants or newborns with congenital heart defects
Exposure (for observational studies): thymectomy
Comparators: individuals not subjected to thymectomy
Outcomes: functioning of the immune system
Study designs: observational studies

### Inclusion and Exclusion Criteria

Only original studies that analyzed any component of the immune system (cells or mediators) in patients with congenital heart defects who had undergone thymectomy during cardiac surgery in the first years of life were included. Studies had to employ a cross-sectional or cohort design, be published in peer-reviewed journals, and available as full text publications. Only papers published in English were included. Case reports, reviews, editorials, and abstracts of congresses were excluded, as well as studies that reported thymectomy in different populations other than subjects with CHD.

### Search Strategy

The databases used for study identification were MEDLINE, EMBASE, LILACS and Scopus. The search strategy included the following key terms: “infant”, “newborn”, “congenital heart defect”, “thymectomy” and “thymus”. For example, the MEDLINE search strategy is detailed in Supplementary Material 1. Additionally, the reference lists of all selected studies were also searched for other sources of information.

### Study Selection and Data Collection

**Figure 1:**
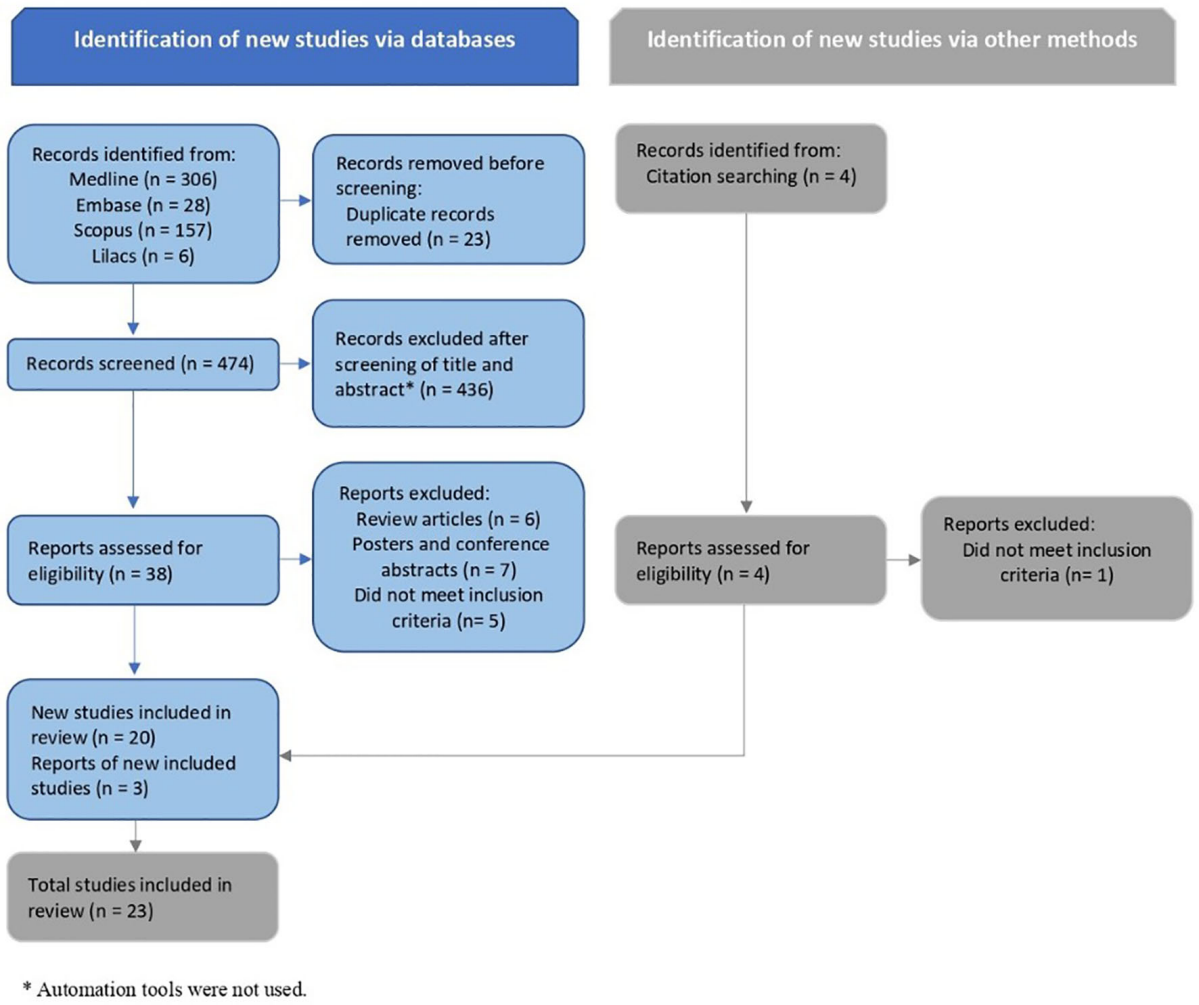
PRISMA flow diagram of records identification, screening and inclusion.

Initially, two authors independently screened titles and abstracts to identify studies for potential inclusion. The full text of these articles was retrieved and reviewed by the same two researchers to ensure compliance with the eligibility criteria. The opinion of a third, independent reviewer was requested in case of disagreement about the inclusion of any study. The selection process for articles was summarized in a flow chart according to the PRISMA recommendations (**Figure 1**). Study details (author, year, and country), study design (type of study, aims, method used for data collection, sample methods, and inclusion/exclusion criteria), participants’ characteristics (number of participants, population characteristics, age at thymectomy, time span since thymectomy, and characteristics of control group), and laboratory analysis (diagnostic methods, cells or mediators analyzed, and the results) were extracted from each article and computed in standard forms specifically designed for data collection.

### Assessment of the Methodological Quality and Risk of Bias

The risk of bias in each study selected for this systematic review was analyzed based on the recommendations of the Newcastle–Ottawa Scale (13). We considered studies with 7-9 stars to have a low risk of bias, studies with 5-6 stars to have a moderate risk of bias, and studies with less than 5 stars to have a high risk of bias.

### Assessment of the Quality of Evidence

The quality of the evidence in this systematic review was assessed according to the Grading of Recommendations Assessment, Development and Evaluation Working Group (GRADE) (14).

## Results

The results of the search are summarized in the study flow diagram (**Figure 1**). Twenty-three studies were included involving 1,446 participants, of which 621 were thymectomized patients and 825 were controls. The sample size ranged from 16 (15) to 193 (16) participants per study, while the number of participants per group ranged from 7 (6) to 154 (16). All chosen studies were published in the last 25 years, between 1996 and 2017, except for one that was published in 1987 (3). All studies included age-matched controls, and eight studies mentioned prospective data collection (3, 9, 17–22). Thymectomy was performed as early as the first days of life up to six years old. The follow-up period ranged from 1 month (20) to 31 years (16) after thymectomy. Most studies reported the presence of a syndrome or genetic disorder (e.g., 22q11 deletion or trisomy 21) as an exclusion criterion (7, 9, 10, 16–27). The main characteristics of the selected studies are shown in **Table 1**.

**Table 1:** Main characteristics of included studies.

Author, year, country	# of patients per group and extension of Tx	Age at Tx	Follow-up	Exclusion criteria
Gudmundsdottir et al., 2017, Sweden (17)	Case: 11 (>90% thymic removal) Control: 11 HS	<6m	18y after Tx	Clinical signs or symptoms suggestive of a syndromic congenital cardiac malformation, including trisomy 21, 22q11.2 deletion syndrome, or CHARGE syndrome
Silva et al., 2017a, Portugal (6)	Case: 7 (total Tx, LT) Control: 20 HS	Early infancy	Median of 24y of age	Not mentioned
Silva et al., 2017b, Portugal (28)	Case1: 8 (total Tx, VLT) Case2: 14 (total Tx, LT) Control: 20 HS	Median of 21m (Case1) and 8m (Case2)	Median of 23y of age (Case1) and 25y (Case2)	Not mentioned
Van den Broek et al., 2017, Netherlands (7)	Case1: 10-27 (total Tx, 1-5y) Case2: 26 (total Tx, >9y) Control1: 10-31 (HS 1-5y) Control2: 11 (HS > 10y)	<1m	1-5y after Tx, 9-29y after Tx (mean 16y)	Clinical signs of infection at time of blood draw and the presence of a syndrome or genetic disorder (e.g., 22q11 deletion, trisomy 21)
Gudmundsdottir et al., 2016, Sweden (9)	Case: 11 (>90% thymic removal) Control: 11 HS	<6m	18m and 18y after Tx	Syndromic cardiac malformation or a known genetic disorder
Van den Broek et al., 2016, Netherlands (10)	Case1: 17 (total Tx, <5y) Case2: 26 (total Tx, >10y) Control1: 19 (HS 1-5y) Control2: 11 (HS > 10y)	<1m	Median of 2.12y of age (Case1) and 15.89y of age (Case2)	Clinical signs of infection at time of inclusion and the presence of a syndrome or genetic disorder (e.g., 22q11 deletion, trisomy 21)
Zlamy et al., 2016, Germany (29)	Case1: 23 (total Tx, <24m) Case2: 12 (total Tx, >24m) Control: 26 HS	Median of 0.2y (Case1) and 5.1y (Case2)	Median of 17.9y of age (Case1) and 17.4y of age (Case2)	Same as Prelog (23)
Elder et al., 2015, USA (18)	Case: 10 (total Tx) Control: 8 (CHD, no Tx)	<1y	> 18y of age	Patients with known or suspected DiGeorge syndrome, current pregnancy, serious infection requiring hospitalization or medication within the prior 3 months, or NYHA class III-IV
Schadenberg et al., 2014, Netherlands (25)	Case: 26: (total Tx) Control: 17 (CHD, no Tx and HS)	<1m	Median of 11.4m of age	Patients with a known syndrome or genetic disorder (eg 22q11 deletion and trisomy 21)
Kurobe et al., 2013, Japan (19)	Case: 17 (total Tx) Control: 15 (partial Tx and no Tx)	<3m	6, 12, 18, 24, 30 and 36m after Tx	Patients with trisomy 21 and chromosome 22q11.2 deletion syndrome
Sauce et al., 2012, France (27)	Case: 25 (total Tx) Control: 20 HS	<2w	Median of 22y of age	Residual cyanosis, transplantation or immunosuppressive therapy, cortisone therapy or hematologic disorders, medication with drugs known to influence blood production in the bone marrow or the immune system
Cao et al., 2011, China (20)	Case1: 20 (small partial Tx) Case2: 15 (sub-total Tx) Control1: 12 (CHD, no Tx) Control2: 25 HS	–	0, 1, 3, 6 and 12m after Tx (TREC level) 1m after Tx (other tests)	History of recent infections, received blood products or immune inhibitors or DiGeorge syndrome
Van Gent et al., 2011, Netherlands (16)	Case: 39 (total Tx) Control1: 102 (HS 0-18y) Control2: 52 (HS 21-39y)	<1.5y	2m – 31y after Tx	Clinical signs of infection at time of blood draw and the presence of a syndrome or genetic disorder (e.g., 22q11 deletion)
Eysteinsdottir et al., 2009, Iceland (30)	Case: 16 (total or partial Tx) Control: 16 HS	Mean of 2.2m	Mean of 14.1y of age	Not mentioned
Prelog et al., 2009, Austria (23)	Case1: 58 (total Tx, <12y) Case2: 43 (total Tx, >12y) Control: 81 HS	Mean of 2.6y (Case1) and 3.2y (Case2)	Mean of 11.1y of age	Residual cyanosis, transplantation or immunosuppressive therapy, cortisone therapy or hematologic disorders, medication with drugs known to influence blood production in the bone marrow or the immune system, allergic disorders, syndromes (eg. trisomy 21 and 22q11 deletion, excluded by genetic screening), vaccination or infections in the last 2-6 weeks prior to taking blood sample
Sauce et al., 2009, France (24)	Case: 25 (total Tx) Control: 90 HS	<2w	Median of 22y of age	Blood transfusion, residual cyanosis, genetic disorder, transplantation, hematologic disorders, immunosuppressive or cortisone therapy, or other medications known to influence the bone marrow or the immune system
Mancebo et al., 2008, Spain (21)	Case: 23 (Tx^a^) Control: 105 HS	<1m	0, 6, 12, 18, 24 and 36m after Tx	Patients with DiGeorge syndrome (by investigating the 22q11.2 chromosomal deletion)
Torfadottir et al., 2006, Iceland (15)	Case: 8 (total or partial Tx) Control: 8 HS	Mean of 2.5m	Mean of 12.1y of age	Not mentioned
Halnon et al., 2005, USA (26)	Case1: 18 (partial Tx) Case2: 11 (total Tx) Control: 26 (CHD, no Tx)	<7y	Mean of 4.7y of age (Case1) and 8.4y of age (Case2)	22q11 chromosomal deletion (by fluorescence in situ hybridization) or recent infections
Eysteinsdottir et al., 2004, Iceland (31)	Case: 19 (total or partial Tx) Control: 19 HS	Mean of 2.6m	Mean of 10.1y of age	Not mentioned
Wells et al., 1998, USA (22)	Case: 25 (Tx^a^) Control: 10 HS	<1m	3 and 12m of age	Patients with DiGeorge syndrome or asplenia
Ramos et al., 1996, Brazil (32)	Case1: 13 (total Tx, <1y) Case2: 10 (total Tx, >1y) Control: 23 (CHD, no Tx)	Mean of 7.9m (Case1) and 2.9y (Case2)	Mean of 5.5y of age (Case1) and 8.3y of age (Case2)	Not mentioned
Brearley et al., 1987, UK (3)	Case: 18 (Tx^a^) Control1: 18 (CHD, no Tx) Control2: 18 HS	<3m	9m – 3y after Tx,	Not mentioned

CHD, congenital heart disease; HS, health subjects; LT, low TRECs; M, months; NYHA, New York Heart Association; Tx, thymectomy; VLT, very low TRECs; W, weeks; Y, years.

a No description of Tx extension.

### Bias Assessment

Detailed descriptions of the risk of bias in the included studies are summarized in **Table 2**. All studies were classified as having a low or moderate risk of bias. All studies had adequate comparability, but all of them failed to report response or follow-up rates.

**Table 2:** Risk of bias of included studies according to the Newcastle–Ottawa Scale.

Author (year)	Selection	Comparability	Outcome/Exposure	Overall quality report
Gudmundsdottir et al. (2017) (17)	3	2	2	7
Silva et al. (2017a) (6)	2	2	2	6
Silva et al. (2017b) (28)	2	2	2	6
Van den Broek et al. (2017) (7)	2	2	2	6
Gudmundsdottir et al. (2016) (9)	3	2	2	7
Van den Broek et al. (2016) (10)	2	2	2	6
Zlamy et al. (2016) (29)	2	2	2	6
Elder et al. (2015) (18)	2	2	2	6
Schadenberg et al. (2014) (25)	3	2	2	7
Kurobe et al. (2013) (19)	2	1	2	5
Sauce et al. (2012) (27)	2	2	2	6
Cao et al. (2011) (20)	2	2	2	6
Van Gent et al. (2011) (16)	2	2	2	6
Eysteinsdottir et al. (2009) (30)	2	2	2	6
Prelog et al. (2009) (23)	2	2	2	6
Sauce et al. (2009) (24)	2	2	2	6
Mancebo et al. (2008) (21)	2	2	2	6
Torfadottir et al. (2006) (15)	2	2	2	6
Halnon et al. (2005) (26)	2	2	2	6
Eysteinsdottir et al. (2004) (31)	2	2	2	6
Wells et al. (1998) (22)	2	2	2	6
Ramos et al. (1996) (32)	2	2	2	6
Brearley et al. (1987) (3)	2	2	2	6

### Clinical Outcomes

Thirteen studies assessed the occurrence of specific diseases, mainly infectious diseases, as well as autoimmune and allergic diseases (**Table 3**). Two studies observed a significantly higher occurrence of the following findings in thymectomized patients compared to controls: hospitalizations (associated with infectious diseases) and mean duration of antibiotic use (19, 20). Four other studies reported the occurrence of infections but did not perform a statistical analysis (3, 9, 15, 23). The remaining seven studies did not identify different clinical manifestations among thymectomized patients and controls (6, 7, 16, 21, 22, 24, 31). Regarding autoimmune and allergic disorders specifically, these conditions were actively evaluated in more recent studies (6, 7, 9), and only one observed a higher occurrence of allergies, but a statistical analysis was not performed due to the small sample size (9). Neoplasias were not described in the selected studies.

**Table 3:** Main results regarding clinical outcomes of included studies.

Author (year)	Thymectomized patients compared to controls	*P* value
Silva et al. (2017a) (6)	No allergy or autoimmunity	–
Van den Broek et al. (2017) (7)	No autoimmunity	–
Gudmundsdottir et al. (2016) (9)	> report of infections and allergies^a^	–
Kurobe et al. (2013) (19)	↑ frequency of hospital admission	<0.02
Cao et al. (2011) (20)	↑ mean duration of antibiotic use (sub-total Tx *vs* controls) 14.1 days (±3.5)^b^ *vs* 9.9 days (±3.1)^b^	<0.01
Van Gent et al. (2011) (16)	No report of infections	–
Prelog et al. (2009) (23)	> report of infections^a^; NSS autoimmunity or cancer	–
Sauce et al. (2009) (24)	NSS hospital admission or infection	–
Mancebo et al. (2008) (21)	NSS infection, allergy, autoimmunity or cancer	–
Torfadottir et al. (2006) (15)	> report of allergies, psoriasis and acute otitis media^a^	–
Eysteinsdottir et al. (2004) (31)	No report of diseases	–
Wells et al. (1998) (22)	NSS hospital admission or infection	–
Brearley et al. (1987) (3)	> report of post-operative infections^a^	–

NSS, not statistically significant; Tx, thymectomy.

a Did not perform statistical analysis.

b Results are shown as mean ± standard deviation in brackets. ↑, higher.

### Numbers of T Cells and Their Subpopulations

Twenty-one studies evaluated total T cells or at least one T cell subpopulation (**Table 4**). Although some of these studies only described percentages of T cells, only absolute cell numbers were considered for the purpose of interpreting pooled results. Regarding total T cells (CD3+), CD4+ and CD8+ T lymphocytes, 10 of 14 studies observed reduced absolute numbers of these cells in thymectomized individuals compared to controls after 0.2-31 years of follow-up (3, 9, 15, 19, 21, 23–26, 31). Three studies performed by the same research group also reported lower numbers of total T cells, CD4+ and CD8+ T cells in the first five years of life, but after five (16) and nine years of life (7, 10), numbers were equivalent to controls. Another study observed lower numbers of these cells only in children who underwent thymectomy in the first year of life but not in those who underwent surgery later (32). Two studies performed a regular follow-up every six months after thymectomy until 36 months and did not detect the typical lymphocytosis (33) that occurs in the first years of life (19, 21).

**Table 4:** Main results regarding numbers of T cells and their subpopulations of included studies.

Author (year)	CD3+	CD4+	CD8+	Treg	Naive T cell	Memory T cell
Silva et al. (2017a) (6)	–	–	–	NSS	↓ CD4, CD8	–
Silva et al. (2017b) (28)	↓^a^ VLT	NSS^a^	–	–	↓ CD4 VLT	–
Van den Broek et al. (2017) (7)	↓ 1-5y; NSS >9y	↓ 1-5y	–	↓ 1-5y; NSS >9y	↓ 1-5y CD4, CD8	↓ 1-5y CD4, CD8
Gudmundsdottir et al. (2016) (9)	–	↓ 18m; 18y	↓ 18m; 18y	↓	↓ CD4, CD8	NSS CD4, CD8
Van den Broek et al. (2016) (10)	↓ 1-5y; NSS >10y	↓ 1-5y; NSS >10y	↓ 1-5y; NSS >10y	–	–	–
Zlamy et al. (2016) (29)	–	–	–	–	↓^a^ CD8	–
Elder et al. (2015) (18)	–	↑^a^	↓^a^	–	NSS^a^ CD4; ↓^a^ CD8	NSS^a^ CD4, CD8
Schadenberg et al. (2014) (25)	–	↓	–	↓	–	–
Kurobe et al. (2013) (19)	↓	↓	↓	↓	–	–
Cao et al. (2011) (20)	NSS^a^	NSS^a^	NSS^a^	–	–	–
Van Gent et al. (2011) (16)	–	↓ <5y; NSS >5y	↓ <5y; NSS >5y	–	↓ <5y; NSS >5y, CD4, CD8	NSS <5y and >5y, CD4, CD8
Eysteinsdottir et al. (2009) (30)	–	–	–	NSS^a^	–	–
Prelog et al. (2009) (23)	–	↓ >12y	–	–	↓ >12y	↓ >12y
Sauce et al. (2009) (24)	–	↓	↓	NSS	↓^a^ CD4, CD8	–
Mancebo et al. (2008) (21)	↓	↓	↓	–	↓ CD4, CD8	NSS CD4, CD8
Torfadottir et al. (2006) (15)	↓	↓	↓	↓	↓ CD4	–
Halnon et al. (2005) (26)	↓	↓	↓	–	↓ CD4	–
Eysteinsdottir et al. (2004) (31)	↓	↓	↓	–	↓ CD4, CD8	↓ CD4, CD8
Wells et al. (1998) (22)	↓^a^	↓^a^	NSS^a^	–	NSS^a^ CD4	NSS^a^ CD4
Ramos et al. (1996) (32)	↓ <1y; NSS >1y	↓ <1y; NSS >1y	↓ <1y; NSS >1y	–	–	–
Brearley et al. (1987) (3)	↓	↓	↓	–	–	–

Absolute numbers of B lymphocytes (7, 9, 19, 21, 31) and NK cells (9, 21, 24, 31, 32) were similar between thymectomized patients and controls.

M, months; NSS, not statistically significant; Tx, thymectomy; VLT, very low TRECs, y, years.

a Analyzed only percentual values. ↑, higher; ↓ lower.

Absolute numbers of naïve and memory T cells were evaluated in 10 studies (6, 7, 9, 15, 16, 21, 23, 26, 28, 31). In all except one study, the numbers of naïve CD4+ and CD8+ T cells were lower in thymectomized patients than in controls throughout the follow-up period. One study reported reduced numbers in the first five years of life, and levels were similar to controls in older patients (16). Of these 10 studies, six analyzed memory T cells (7, 9, 16, 21, 23, 31), three of which observed decreased numbers in thymectomized individuals (7, 23, 31), whereas the other three did not detect any difference between the groups (9, 16, 21).

Regulatory T cells (Treg cells) were analyzed in seven studies, of which four (9, 15, 19, 25) reported reduced numbers in thymectomized individuals compared to controls, and two (6, 24) obtained similar results when comparing the groups. Only one study observed an initial decrease in the first five years of life with a subsequent equivalence to controls in individuals older than 9 years (7). The expression levels of CTLA-4 and CD39, markers associated with a suppressive functional phenotype within memory Tregs, were also similar (6).

### Thymic and T Cell Functional Markers

T cell receptor excision circles (TRECs) values were lower in thymectomized patients than in controls in eight of nine studies (6, 9, 16, 20, 21, 23, 26, 28, 29) (**Table 5**). One study observed decreased values in the first five years of life with a subsequent equivalence to controls afterward (16). The proportions and absolute counts of CD31 on CD4+ and Treg cells, which is also considered a marker of recent thymic emigrants, was consistently reduced in thymectomized patients in all studies (9, 10, 24, 25, 28, 29), except for one in which patients were equivalent to controls (7). Furthermore, one study described an inverse correlation between the proportion of CD31 on naïve CD4+ T cells and the expression of Ki67 in the same subpopulation (27).

**Table 5:** Main results regarding numbers of TRECs of included studies.

Author (year)	Thymectomized patients compared to controls	*P* value
Silva et al. (2017a) (6)	0.52* (0.05-1.8)^a^ *vs* 17.2* (4.01-39.3)^a^	0.001
Silva et al. (2017b) (28)	↓ VLT and LT *vs* controls	<0.0001 (VLT); 0.004 (LT)
Gudmundsdottir et al. (2016) (9)	Nondetectable values in 10 out of 11 individuals	<0.01
Zlamy et al. (2016) (29)	Detectable values in 14.7% *vs* 82.4%	0.001
Cao et al. (2011) (20)	↓ levels after sub-total Tx compared to levels before surgery	<0.01
Van Gent et al. (2011) (16)	↓ numbers (in the first 5 years after Tx) NSS numbers (5 years after Tx)	<0.001
Prelog et al. (2009) (23)	1275** (525-3616)^a^ *vs* 5410** (2056-11,194)^a^ (in individuals > 12 years old)	<0.001
Mancebo et al. (2008) (21)	↓ levels after 6, 12, 18, 24 and 36 months after Tx	<0.01
Halnon et al. (2005) (26)	↓ numbers in subjects who had undergone surgical procedure compared to those who had no prior surgery	<0.0001 (no residual thymus); <0.001 (with residual thymus)

LT, low TRECs; NSS, not statistically significant; Tx, thymectomy; VLT, very low TRECs.

a Results are shown as median and ranges in brackets.

*TRECs/μL.

**TRECs per 10^5^ CD4^+^CD45RA^+^CD62L^+^ T cells. ↓, lower.

Thymectomized individuals had a decreased diversity of the T cell receptor (TCR) repertoire compared to controls in all six studies in which this analysis was performed (9, 17, 24, 28–30). One additional study also reported a skewed repertoire of TCR Vβ families in thymectomized patients (21).

Lymphocyte proliferation was evaluated using different methods. Ki67 expression was increased in thymectomized patients compared to controls in five of six studies (7, 16, 23, 27–29) (**Table 6**). One study observed an initial increase in Ki67 expression in the first five years of life, with values similar to controls observed after nine years of age (7). A similar trend of results was observed for other markers and cell numbers described in two studies performed by the same research group (10, 16). Significantly shorter relative telomere lengths of T cells were also detected in thymectomized individuals in two studies (9, 29). Six other studies analyzed the T cell proliferative response to different mitogens, such as phytohemagglutinin (PHA) (3, 19–22, 31), anti-CD3 (21) and phorbol myristate acetate (PMA) plus ionomycin (21). The mitogen-induced proliferation of T cells was similar among the groups in all except for one study that observed a diminished response to PHA in thymectomized individuals (3).

**Table 6:** Main results regarding expression of Ki67 of included studies.

Author (year)	Thymectomized patients compared to controls	*P* value
Silva et al. (2017b) (28)	↑ % Ki67^+^ in naive CD4^+^ VLT *vs* controls and VLT *vs* LT	<0.0001 (VLT *vs* controls) 0.0007 (VLT *vs* LT)
Van den Broek et al. (2017) (7)	↑ % Ki67^+^ in CD3^+^ (1-5 years after Tx) NSS % Ki67^+^ in CD3^+^ (9 years after Tx)	<0.05
Zlamy et al. (2016) (29)	↑ % Ki67^+^ in CD127^+^ in memory CD8^+^	0.02
Sauce et al. (2012) (27)	↑ % Ki67^+^ in naive CD4^+^	0.01
Van Gent et al. (2011) (16)	↑ % Ki67^+^ in naive CD4^+^ and CD8^+^	0.03 (CD4^+^); 0.01 (CD8^+^)
Prelog et al. (2009) (23)	% Ki67^+^ in naive CD4^+^ (in individuals > 12 years old) 0.4 (0.1-1.7)^a^ *vs* 0.2 (0.1-0.8)^a^	<0.05

LT, low TRECs; NSS, not statistically significant; Tx, thymectomy; VLT, very low TRECs.

a Results are shown as median and ranges in brackets. ↑, higher.

Different markers were also assessed, such as the presence of CD127, Bcl-2, CD103, PD1 and CD57 and the absence of CD27 and CD28 (see the details in **Table 7**). The proportion of T cell subpopulations expressing markers typically associated with lymphocyte exhaustion (PD1+) and senescence (CD57+, CD27- and CD28-) was similar between the groups (18, 24).

**Table 7:** Main results regarding levels of other markers of included studies.

Author (year)	Thymectomized patients compared to controls	*P* value
Silva et al. (2017a) (6)	NSS CTLA-4, CD39+	–
Silva et al. (2017b) (28)	↓ % and absolute count CD31+ in naïve CD4+ of VLT ↑ Bcl-2 in naïve CD4+ of VLT and LT	<0.0001 (%) and 0.0001 (absolute) 0.0187 (VLT) and 0.0487 (LT)
Van den Broek et al. (2017) (7)	Normal % CD31+ in naive Tcells >9 years after Tx	No comparison to controls
Gudmundsdottir et al. (2016) (9)	↓ % CD31+ in naive CD4+ (55% *vs* 81%)	0.034
Van den Broek et al. (2016) (10)	↓ % CD31+ in naïve CD4+ 1-5 years and >10 years after Tx	<0.05
Zlamy et al. (2016) (29)	↓ % CD31+ in CD8+, ↓ % CD127+ of naïve CD8+, ↑ % CD103+CD8+ in lymphocytes	0.03, 0.04, 0.05
Elder et al. (2015) (18)	NSS % PD1+, CD57+, CD27-, CD28- in CD8+	–
Schadenberg et al. (2014) (25)	↓ % CD31+ in Treg cells	<0.05
Sauce et al. (2012) (27)	Inverse correlation between % of CD31+ and Ki67 in naïve CD4+	<0.0001
Eysteinsdottir et al. (2009) (30)	NSS % CD127+ in Treg cells	–
Sauce et al. (2009) (24)	↓ % CD31+ in naive CD4+, NSS % CD57+ in memory T cells	<0.0001

LT, low TRECs; NSS, not statistically significant; Tx, thymectomy; VLT, very low TRECs. ↑, higher; ↓ lower.

Serum levels of cytokines and chemokines were quite diverse (**Table 8**). IL-7 was the cytokine most commonly evaluated, and its level was increased in thymectomized individuals compared to controls in three studies (16, 21, 27), reduced in one study (28) and similar in another study (29). Furthermore, lower proportions of CD127+ cells among naïve CD8+ T cells were observed in thymectomized individuals than in controls (29). Other cytokines and chemokines were also analyzed, and the following differences in their serum levels were observed in thymectomized individuals compared to controls: (i) increased IFN-γ, IL-17, IL-13, TNF-α, CXCL13, IL-8, IL-1β and eotaxin; (ii) decreased IL-8; and (iii) similar IL-21, IL-2, and IL-4 (7, 10, 20, 24).

**Table 8:** Main results regarding levels of cytokines of included studies.

Author (year)	Thymectomized patients compared to controls	*P* value
Silva et al. (2017b) (28)	IL-7 (LT *vs* control) 12.8* (5.3-16.2)^a^ *vs* 15.0* (6.5-23.3)^a^	<0.05
Van den Broek et al. (2017) (7)	↑ IFN-γ, IL-17, IL-13, TNFα, CXCL 13; NSS IL-21 (1-5 years after Tx)	<0.05
Van den Broek et al. (2016) (10)	↓ IL-8; NSS IL-2 (1-5 years after Tx)	<0.05
Zlamy et al. (2016) (29)	NSS IL-7	–
Sauce et al. (2012) (27)	↑ IL-7	0.03
Cao et al. (2011) (20)	NSS IL-2, IL-4, IFN-γ	–
Van Gent et al. (2011) (16)	↑ IL-7 (in the first 2.5 years after Tx)	0.012
Sauce et al. (2009) (24)	↑ IL-1β, IL-8 and eotaxin	<0.005
Mancebo et al. (2008) (21)	IL-7 (1 year and 2 years after Tx *vs* control) 11.1* (±9.9)^b^ and 13.9* (±11.4)^b^ *vs* 3.8* (±3.1)^b^	<0.01

NSS, not statistically significant; Tx, thymectomy.

a Results are shown as median and ranges in brackets.

b Results are shown as mean ± standard deviation in brackets.

*pg/ml. ↑, higher; ↓ lower.

### Humoral Immunity and Autoantibodies

Levels of different immunoglobulins were analyzed in seven studies (3, 7, 15, 19, 21, 22, 31) (**Table 9**). Total IgG (7), IgA (3, 31) and IgG1 (31) levels were lower in thymectomized patients than in controls, whereas similar levels within the normal range were observed between the groups for the following immunoglobulins: IgG (3, 21, 31), IgM (3, 7, 21, 31), IgA (21) and IgE (31). Specific IgG antibody titers to measles and rubella viruses were lower in thymectomized individuals than in controls in one study (19), while specific antibody levels were equivalent between the groups for the following antigenic preparations: tetanus (3, 15, 22), measles (15), mumps (15) and diphtheria (3). Only one study evaluated the antibody response to T-independent antigens (pneumococcal polysaccharides) and did not observe differences between the groups (3).

**Table 9:** Main results regarding levels of immunoglobulins of included studies.

Author (year)	Thymectomized patients compared to controls	*P* value
Van den Broek et al. (2017) (7)	↓ IgG; NSS IgM	<0.05
Kurobe et al. (2013) (19)	↓ IgG to measles and rubella	<0.05 and <0.01
Mancebo et al. (2008) (21)	IgG, IgA and IgM within normal range	No comparison to controls
Torfadottir et al. (2006) (15)	NSS IgG to tetanus, measles and mumps	Data not shown
Eysteinsdottir et al. (2004) (31)	↓ IgA e IgG1; NSS IgM, IgG, IgE	<0.05
Wells et al. (1998) (22)	NSS IgG to tetanus	–
Brearley et al. (1987) (3)	↓ IgA; NSS IgG and IgM NSS IgG to tetanus and diphteria	<0.01

NSS, not statistically significant. ↓, lower.

Autoantigens microarrays were used in two studies and detected 125 (6) and 911 (7) autoantigens. Van den Broek et al. (7) found 68 autoantibodies that were altered in thymectomized subjects younger than 5 years compared to controls. Clustering analysis detected two different profiles of autoantibody reactivity between the groups: Cluster 1 showed a lower autoantibody intensity in thymectomized children (mostly IgM autoantibodies), whereas Cluster 2 showed increased autoantibody intensity in thymectomized children (mostly IgG isotypes). Silva et al. (6) analyzed IgG reactivity to distinct autoantigens and observed higher expression of 18 autoantibodies that have been previously identified in patients with systemic lupus erythematosus in thymectomized individuals than in controls. Moreover, a cluster of IgG autoantibodies clearly associated with autoimmune liver disease was also identified in thymectomized individuals. Rheumatoid factor (RF) (23, 31), antinuclear antibody (ANA) (23, 31) and autoantibodies against thyroglobulin, parietal cells and pancreatic islet cells (15) were not detected in thymectomized patients in three studies, while ANA and/or antineutrophil cytoplasmic antibody (ANCA) were detected in 16 of 26 thymectomized individuals older than nine years compared to three of nine controls (7).

## Discussion

The present study performed the first systematic review of the literature describing the consequences of early thymectomy on long-term functioning of the immune system and observed that children who underwent thymectomy in the first years of life have reduced numbers of total T cells, CD4+, CD8+, naïve T cells, TRECs and CD31, a decreased diversity of the TCR repertoire and an increased expression of Ki67 and IL-7 compared to controls in the first five years after surgical procedure as an overall trend. These alterations exhibited a long-term persistence in most studies. However, the numbers of memory T cells and Treg cells differed across the selected studies.

Notably, this marked reduction in lymphocyte populations occurs at a particular age when humans typically present lymphocytosis (33). Interestingly, the numbers of T cells and subpopulations were not consistently decreased in thymectomized patients in all studies but varied according to the specific cell subtype and time after surgery. For instance, regarding the age at follow-up, thymectomized children regularly presented reduced numbers of total T cell, CD4+, CD8+ and naïve T cells up to five years after surgery, whereas older subjects presented divergent results. Studies with longer follow-up, including patients in the second and third decades of life, revealed that thymectomized patients either exhibit lower or similar levels of these cells. Some possible explanations for these different results have been proposed, such as the amount of thymus removed and the age at thymectomy. Most studies stated that total or > 90% removal of the thymus was performed; however, some studies included total and partial thymectomy in the same group and nevertheless observed persistently reduced levels of total T cells, CD4+, CD8+ and naïve T cells. Since thymectomy was not the reason for the operation but was only performed for ease of surgical access to the heart and great vessels, and most studies were retrospective, it was not possible to guarantee the completeness of the procedure. Cardiac surgeons usually try to remove the whole thymus in an attempt to avoid bleeding, but are not usually able to exclude *in situ* remains of residual cervical extensions of thymic tissue. Importantly, modern cardiac surgery has been more prone to avoid removal of any thymic tissue once an adequate view of the surgical field is obtained by displacing the thymus, except when manipulation of great vessels is required (Jatene MB, personal communication). Thymic epithelial progenitors in postnatal cultured thymus tissue are responsible for the development of functioning allografts after thymus transplantation in infants with complete DiGeorge anomaly (34). Therefore, if sufficient numbers of these progenitors are preserved during thymectomy, then these cells might be responsible for the regrowth of functionally competent thymic tissue (16). In addition to compensatory expansion of residual thymic tissue, ectopic rudiments may also proliferate. Indeed, thymic tissue was observed in MRI scans of some patients who underwent thymectomy during the first years of life, but this fact does not confirm that the tissue is active and capable of thymopoiesis (16).

Regarding the age when thymectomy was performed, substantial heterogeneity was observed among the selected studies, ranging from newborns younger than two weeks of age up to six-year-old children. It was not possible to establish a clear association between the age at thymectomy and subsequent numbers of T cells and subpopulations. Some studies specifically examined this aspect. In 1996, Ramos et al. divided thymectomized children into two groups according to age at the time of surgery (less than one year old and older than one year old) and observed that children who underwent thymectomy in the first year of life had fewer numbers of total T cells, CD4+ and CD8+ than controls, whereas those submitted to surgery after the first year of life had similar levels to controls (32). However, in 2009, Prelog et al. did not observe any correlation between the age at thymectomy and the numbers of total, naïve and memory CD4+ T cells (23).

On the other hand, Treg cells and memory T cells did not exhibit homogeneous results across the studies, even in young children. The preserved Treg compartment observed in thymectomized individuals in some studies might be explained by the precocious release of Treg cells in early life (35, 36) and the role of peripheral mechanisms that guarantee Treg homeostasis (37). One of these mechanisms is mediated by IL-7, which was detected at increased levels in thymectomized patients in some studies, although similar levels of IL-2, the cytokine that stimulates peripheral proliferation of Tregs, was observed. Moreover, a trend toward the effector/memory phenotype, probably at the expense of naïve T cells, was reported in some studies and has been observed in individuals with other persistent lymphopenic conditions, such as trisomy 21 and aging (38–40). Furthermore, in 2016, van den Broek et al. described an increased proportion of T memory stem cells (Tscm) in thymectomized individuals, which are a rare subset of memory lymphocytes endowed with the stem cell-like ability to self-renew and the multipotent capacity to reconstitute the entire spectrum of memory and effector subsets (41). They hypothesized that the increased Tscm compartment after thymectomy might be compensatory for the reduced number of naïve T cells and can promote the expansion of the effector/memory subsets to levels similar to controls (10). However, the authors were unable to confirm a definite contribution, as absolute numbers of Tscm were decreased in thymectomized patients, despite their increased proportion in CD4+ cells (10).

One of the best methods to assess thymic output in humans is the direct analysis of the TRECs content of a T cell population, and CD31 is a cell surface marker expressed preferentially by naïve TREC-rich T cells that have undergone a low number of cell divisions (42–44). Additionally, obtaining an accurate representation of the peripheral TCR repertoire is of greatest utility when measuring thymic function, as TCR repertoire diversity reflects both the capacity of the thymus to generate naïve T cells and the cumulative responses of T cells to antigen challenges in the periphery (42). All these markers of thymic activity were remarkably compromised in thymectomized individuals, indicating that as thymic export stops, T cell homeostasis is modified and premature signs of immune aging become evident.

Interestingly, Silva et al. (28) divided thymectomized patients into two groups according to TRECs levels and showed that adults with some thymic activity (TRECs levels within the range of age-matched controls, although significantly lower) were able to preserve the naïve CD4+ and CD31+ T cell compartments and the diversity of the TCR repertoire, in contrast to thymectomized adults lacking thymic activity (28). Furthermore, subjects with no evidence of thymic tissue during a visual inspection were more likely to have undetectable levels of TRECs, whereas those with thymic tissue present had higher TRECs levels that were often normal (26). These findings indicate that T cell compartment activity may persist through both peripheral mechanisms and thymus regeneration, thus reinforcing the recommendation to preserve some thymic tissue during cardiac surgery.

The proliferation rates of peripheral T cells and subpopulations have been assessed by measuring levels of the cell-cycling marker Ki67 (45), which was consistently expressed at higher levels in thymectomized children in the first five years of life. Additional findings, such as the inverse correlations between (i) the expression of CD31 and Ki67 on naïve CD4+ T cells (27), (ii) TRECs numbers and the expression of Ki67 on naïve CD4+ T cells, and (iii) naïve CD4+ T cell counts and the expression of Ki67 on T cells (23), provide further insights into the mechanisms of homeostatic regulation of the naïve T cell compartment. Decreased numbers of naïve CD4+ T cells may induce the peripheral homeostatic proliferation of these cells, but it is still unsatisfactory to compensate for thymic depletion. Shortened telomeres, markers of the replicative history of T cells, provide advanced evidence of increased peripheral naïve T cell proliferation. The signaling pathway inducing the peripheral homeostatic proliferation of naïve T cells might be mediated by IL-7, a cytokine that plays an important role in survival and proliferation of the naïve T cell pool (46), which was shown to be increased in thymectomized patients and inversely correlated with naïve CD4+ T cell numbers (16).

Regarding humoral immunity, no effect on the number of B lymphocytes was observed. However, quite diverse results for total and specific immunoglobulin levels were reported across the studies, with a trend toward normal serum Ig levels.

A broad analysis of highly sensitive autoantibody identification through autoantigens microarray technology was performed in two recent studies that observed different autoreactivity profiles and higher titers of distinct autoantibodies in thymectomized individuals. Neither of the studies described the occurrence of clinical autoimmune disease, and both hypothesized that the preserved Treg compartment might be responsible for inhibiting the development of signs of autoimmune disease (6, 7).

Notably, none of the alterations in the homeostasis of the immune system of thymectomized individuals consistently translated into clinical manifestations in the studies included in this review. Only two reports documented a significantly higher occurrence of infectious outcomes (hospitalizations associated with infectious diseases and mean duration of antibiotic use), and isolated studies described other infectious and allergic diseases. Several factors may help us understand this finding. Infections may occur after the complex thymectomy surgery, and recall bias may affect the reporting of symptoms, particularly in subjects who have had surgery and an increased frequency of medical visits compared to those who did not undergo a surgical procedure. Additionally, the number of subjects in most studies is relatively small and may be inadequately powered to detect differences. On the other hand, a recent large nationwide population-based cohort study performed in Sweden observed differences in the risks of certain autoimmune diseases, cancer, infections, and asthma following early thymectomy, but the authors were unable to ascertain a causal mechanism because of the observational nature of the study (47). Clinical outcomes that have not yet been observed are likely to occur in the upcoming years since corrective cardiac surgery in young children only became a successful practice four decades ago, hampering longer follow-up studies. These patients may be at higher risk of suffering earlier from age-related conditions, such as autoimmunity and cancer, and may exhibit a poor response to new antigens, such as vaccine antigens.

Drawing a parallel between the population of thymectomized individuals studied here and thymectomy performed in patients with other clinical conditions might be enlightening. For instance, patients with myasthenia gravis (MG) treated with thymectomy have been previously studied, and similar results have been observed in the long term: a significant reduction in the number of TRECs, lower numbers of naïve CD4+ and CD8+ T cells along with an increased proportion of memory CD4+ T cells (48), decreased T cell counts and a reduced TCR repertoire (49). Although this comparison must be carefully interpreted because patients with MG are mainly adults and have differences in immunological maturity, converging findings from both populations strengthen the evidence for premature immunosenescence in the T cell compartment of thymectomized individuals.

The present study has some limitations that are mostly related to the heterogeneity of the selected studies in terms of the following aspects: age when thymectomy was performed, presence or absence of residual thymus, time span since thymus removal and analysis of different markers for T cell subpopulations. Furthermore, most studies included a small number of participants in each group, which precluded some statistical analyses with adequate power. Regarding the analysis of potential bias, the risk was considered low/moderate, which represents a relatively adequate methodological quality of the included studies.

Nevertheless, it was possible to gather the current evidence for the consequences of performing thymectomy in patients undergoing cardiac surgery in early childhood on the functioning of the immune system. An unequivocal effect on essential compartments of the immune system that are directly dependent on thymic output, such as naïve T cells, TRECs, CD31+ T cells and TCR repertoire diversity, was observed, whereas other elements, such as Tregs and memory T cells, were preserved in some studies. These modifications suggest early immunosenescence, indicating that these patients might be at increased risk of developing diseases such as autoimmunity, neoplasms, and even severe infections due to impaired responses to new antigens in the future. However, it is not clearly determined whether the magnitude of the effect is related to the age at thymectomy, the amount of thymus removed or the time elapsed after surgery. These issues may be overcome if TRECs are used as a measure of continuous thymic output. Further research is needed to address these specific topics. As successful cardiac surgery in young children has only been possible in the last four decades, the effects of thymectomy on clinical and laboratory parameters might be more prominent in the future. Based on the numerous data compiled, the main lessons they can currently teach us are that the immune responses of thymectomized patients should be observed and a cautious approach is to preserve some thymic tissue in infants and young children undergoing corrective cardiac surgery in order to promote long and healthy survival. Our findings reinforce the recent trend to avoid thymic removal whenever possible, which is expected to be consistently incorporated into operative techniques. Meanwhile, alternative solutions should be studied, such as the autograft of thymus fragments.

## Data Availability Statement

The original contributions presented in the study are included in the article/**Supplementary Material**. Further inquiries can be directed to the corresponding author.

## Author Contributions

NC, MC-S, and MJ designed the study. NC and MD built the research strategy and performed data extraction. NC, PP, and MC-S analysed the results and wrote the manuscript. MD and MJ reviewed the manuscript. All authors had full access to the study data. All authors contributed to the article and approved the submitted version.

## Funding

Fundação de Amparo à Pesquisa do Estado de São Paulo (FAPESP), grant number 2014/50489-9.

## Conflict of Interest

The authors declare that the research was conducted in the absence of any commercial or financial relationships that could be construed as a potential conflict of interest.

## Publisher’s Note

All claims expressed in this article are solely those of the authors and do not necessarily represent those of their affiliated organizations, or those of the publisher, the editors and the reviewers. Any product that may be evaluated in this article, or claim that may be made by its manufacturer, is not guaranteed or endorsed by the publisher.
