# Supplementary material for: Early Thymectomy Is Associated With Long-Term Impairment of the Immune System: A Systematic Review
Source: Front Immunol. 2021 Nov 25;12:774780. doi: 10.3389/fimmu.2021.774780 (PMC8656688; doi:10.3389/fimmu.2021.774780)
Supplement: Supplementary file 1 [file DataSheet_1.docx]

| **Search strategy - MEDLINE (through Pubmed - advanced search)** |
| --- |
| #1 "Infant"[Mesh] OR Infants OR "Infant, Newborn"[Mesh] OR "Infants, Newborn" OR "Newborn Infant" OR "Newborn Infants" OR Newborns OR Newborn OR Neonate OR Neonates  #2 "Heart Defects, Congenital"[Mesh] OR "Congenital Heart Defect" OR "Defect, Congenital Heart" OR "Heart, Malformation Of" OR "Defects, Congenital Heart" OR "Heart Abnormalities" OR "Heart Defect, Congenital" OR "Abnormality, Heart" OR "Abnormalities, Heart" OR "Heart Abnormality" OR "Congenital Heart Defects"  #3 "Thymectomy"[Mesh] OR Thymectomies OR "Thymus Gland"[Mesh] OR "Gland, Thymus" OR "Glands, Thymus" OR "Thymus Glands" OR Thymus |
